# Demographic Shortcuts Account for Most of the Apparent Accuracy of Four-Class Gait-Based Differential Diagnosis in Neurodegenerative Disease: An Interpretability-Driven Audit of Machine Learning on a Public Gait Benchmark

**DOI:** 10.3390/bioengineering13091092

**Published:** 2026-09-20

**Authors:** Zheng Wang, Yitong Xia, Guifeng Zhang

**Affiliations:** 1 Institute of Neuropathology, University Hospital RWTH Aachen, 52074 Aachen, Germany; 2 Department of Oral Medicine, Jining Medical University, Jining 272000, China; xyt040920@163.com

**Keywords:** gait analysis, neurodegenerative disease, differential diagnosis, machine learning, confounding, shortcut learning, explainable artificial intelligence, Huntington’s disease, stride-interval dynamics, external validation

## Abstract

Machine-learning gait studies often report high accuracy for separating neurodegenerative diseases, but rarely test whether this reflects pathology or the demographic differences that accompany disease in convenience cohorts. We audited the PhysioNet Gait in Neurodegenerative Disease Database (63 subjects: controls, Parkinson’s disease [PD], Huntington’s disease [HD], amyotrophic lateral sclerosis [ALS]), classifying 37 stride-rhythm features under subject-disjoint nested leave-one-subject-out cross-validation. A random forest reached a macro-averaged one-vs-rest AUROC of 0.832 (95% CI 0.759–0.904; permutation *p* = 0.001). Four complementary audits—demographic-only baselines, confound adjustment, feature-family ablation, and age matching—show that this overstates gait-specific discrimination: five anthropometric variables alone reached an AUROC of 0.812, statistically indistinguishable from the gait model (*p* = 0.701), and the de-confounded gait model (0.755) fell further to 0.585 once age was matched across groups. HD was the exception, essentially unaffected by adjustment (0.812 → 0.804) and carried by variability and long-range-correlation measures. In an independent, age-matched cohort recorded with a different sensor (*n* = 110, PD versus control only), the same vulnerability reappeared through a different variable: the gait model (0.828) showed no statistically significant difference from walking speed alone (0.746, *p* = 0.11), while age and anthropometry fell to chance; this cohort contained no HD subjects, so the HD finding remains externally unvalidated. The shortcut vulnerability generalises across cohorts; the demographic variable that drives it does not. Four-class differential diagnosis is not demonstrated on this benchmark; HD gait arrhythmicity is the one signal that withstands internal sensitivity analysis. We recommend that accuracy claims from small, demographically imbalanced gait cohorts be accompanied by this kind of audit.

## 1. Introduction

Quantitative gait analysis is an appealing route to objective markers of neurodegenerative disease. Walking is impaired early in Parkinson’s disease (PD), Huntington’s disease (HD) and amyotrophic lateral sclerosis (ALS) [1,2,3,4]; it can be instrumented cheaply with footswitches or inertial sensors; and the resulting time series carry structure that visual clinical rating cannot resolve. Two decades of work have established that stride-interval fluctuations are not random noise but exhibit long-range correlations that degrade with age and disease, and that the magnitude and temporal organisation of stride-to-stride variability differ between patient groups and controls [5,6,7,8,9].

This matters clinically because the early differential diagnosis of parkinsonian and other neurodegenerative syndromes remains difficult even for specialists [10,11,12], motivating objective instrumented markers [13,14,15,16,17,18,19,20]. Recent staging-oriented deep-learning frameworks extend this trend further, for example combining automated three-dimensional gait-feature extraction with Hoehn–Yahr stage prediction [21] or fusing multimodal imaging and clinical priors for the same purpose [22]. Reported accuracies are often high, and a substantial part of this literature treats such numbers as evidence that disease-specific gait signatures exist and are machine-readable, although we are not aware of a systematic review that has quantified how widespread this interpretation is. In many of these studies, an assumption is left unexamined: that the classifier is reading motor pathology rather than something else that happens to differ between the diagnostic groups in the dataset used.

That assumption is fragile in exactly the datasets on which such studies are performed. Clinical convenience cohorts are not demographically balanced: patients are older than the volunteers recruited as controls, disease severity co-varies with walking speed, and walking speed in turn sets the absolute duration of every gait phase. Because stride, swing, stance and double-support durations are all monotone functions of speed [23,24,25], and because long-range stride correlations and variability magnitude likewise depend on age and speed [23,24,25,26,27], a large fraction of any conventional gait-feature set is a proxy for how fast the subject walked and, through age-related slowing, for how old the subject was. Age, disease and walking speed therefore co-vary in every such cohort, so a classifier trained on these features can achieve strong cross-validated performance while encoding little more than the demographic composition of the cohort—the phenomenon described in machine learning as shortcut learning, in which a model exploits a spurious cue that is predictive within the dataset but does not correspond to the concept the task intends to measure [28]. Demonstrating disease-specific discrimination consequently requires more than a high cross-validated score with subject-disjoint folds: it requires validation designs appropriate to small samples, where optimistic bias and unstable estimates are well documented [29,30,31,32]; it requires showing that the score is not reproducible from demographics alone; and it requires showing that it survives explicit adjustment for the confounds and persists when the confound is removed by design rather than by regression.

The present study performs that examination on the most widely used public benchmark in this area, the PhysioNet Gait in Neurodegenerative Disease Database, which contains bilateral footfall recordings from patients with PD, HD and ALS together with healthy controls [7,33]. Our aim is deliberately not to maximise four-class accuracy. It is to establish how much of the accuracy that a careful, leakage-free pipeline achieves on this dataset is attributable to gait pathology, and how much is attributable to age and walking speed. We therefore (i) build a conventional four-class model under strictly subject-disjoint nested cross-validation and verify it against a permutation null; (ii) compare it against classifiers that use only demographic and anthropometric variables and no gait data at all; (iii) quantify the cost of removing the confound by two independent routes, within-fold residualisation on age and speed, and ablation of the speed-dependent feature family; (iv) attribute the model’s decisions per disease class using out-of-fold Shapley additive explanations (SHAP) values, including the individual subjects it gets wrong; and (v) test whether the result survives age matching and whether it has converged at this sample size.

The outcome reframes what this benchmark can support. The conventional model performs well and is unambiguously better than chance, yet its advantage over two demographic numbers is not statistically reliable, and the de-confounded model is indistinguishable from those two numbers. One signal does survive every audit: HD gait arrhythmicity, expressed through stride-interval variability and degraded long-range correlation rather than through absolute slowness. We report the negative and positive findings together and provide the audit protocol as a reusable component of small-cohort gait studies.

## 2. Materials and Methods

### 2.1. Data Source and Cohort

We used the PhysioNet Gait in Neurodegenerative Disease Database (gaitndd, version 1.0.0), a publicly available archive of bilateral foot-force recordings from patients with PD, HD and ALS and from healthy controls [7,33]. The database contains 64 records. All 258 constituent files were downloaded and verified against the published SHA-256 manifest; every checksum matched.

Signal specifications were read from the WFDB header of every record rather than assumed. All 64 records were uniform: two channels (left-foot and right-foot force-sensitive resistor, FSR), sampling frequency 300 Hz, 90,000 samples corresponding to 300.0 s of walking, format 212 with 12-bit resolution; records differed only in analogue-to-digital gain (1000 or 3000). Each record is accompanied by a derived time-series file containing thirteen per-cycle quantities: elapsed time, left and right stride interval, swing and stance interval in seconds and as a percentage of the stride cycle, and double-support time in seconds and as a percentage.

Subject metadata (group, age, height, weight, sex, walking speed, disease duration or severity) were parsed from the distributed description file. Three defects in that file were identified and corrected: the ALS records carry a group label inconsistent with the other groups, so diagnostic labels were derived from the record-name prefix instead; one HD record is wrapped across two physical lines and was rejoined; and several fields contain a literal missing-value token, which was coerced to a missing value and logged. After correction, walking speed was missing for three of the 64 records (hunt20, als4, als5) and weight for one (als13); because hunt20 was subsequently excluded (Section 2.2), the 63-subject analysis cohort has walking speed missing for two subjects and weight for one.

### 2.2. Quality Control and Exclusions

The first and last five stride cycles of every record were discarded as gait-initiation and gait-termination transients. The remaining cycles were required to have bilateral stride intervals within a physiologically plausible range of 0.3–3.0 s; surviving outliers were removed with a Hampel rule based on the median absolute deviation (*k* = 3, scale factor 1.4826) applied to each foot’s stride-interval series.

One record (hunt20, HD) was excluded in full. Its right-foot FSR failed at source: all 238 right-foot stride intervals were physiologically implausible and constant at 42.91 s, right stance was reported as 41.93 s, and 237 of 238 double-support values were negative. Walking speed was also missing for this subject. A screen of all 64 records confirmed that this was the only case of systemic sensor failure; other flagged records contained one to five isolated outlier cycles that the Hampel rule handled. Excluding the record preserves bilateral asymmetry and double-support features across the whole cohort, which a single-limb fallback would have forced us to abandon.

The final analysis cohort therefore comprised 63 subjects (16 control, 15 PD, 19 HD, 13 ALS); note that the HD group contains 19 records, not the 20 distributed. Across these 63 subjects, 14,922 raw stride cycles were reduced to 14,292 by transient trimming and to 13,440 by artefact rejection, i.e., 6.0% of post-trimming cycles were rejected as artefacts and 9.9% of raw cycles were removed in total (median 225 analysed cycles per subject).

### 2.3. Feature Extraction

Thirty-seven subject-level features were computed from the cleaned bilateral series and assigned a priori to one of four families, a grouping that is central to the audit:

- Absolute (9 features)—mean stride, swing and stance interval; mean swing and stance percentage; mean double-support duration and percentage; swing-to-stance ratio; cadence. These are monotone functions of walking speed and are the confound-prone family.
- Variability (12 features)—standard deviation, coefficient of variation (CV) and interquartile range of the stride, swing and stance intervals; CV of swing and stance percentage; CV of double-support duration.
- Nonlinear dynamics (10 features)—lag-1 autocorrelation and detrended-fluctuation-analysis (DFA) scaling exponent α of the stride, swing and stance interval series [34,35]; sample entropy of the stride-interval series; and three spectral descriptors of that series (fraction of power below 0.1 cycles^−1^, normalised spectral entropy, dominant frequency).
- Asymmetry (6 features)—left–right asymmetry index of the stride, swing and stance interval and of swing and stance percentage; Pearson correlation between left and right stride intervals.

### 2.4. Feature Sets: Full and De-Confounded

Every analysis was run on two feature sets, specified before modelling:

- FULL—all 37 features.
- DECONF—the 28 features remaining after removing the entire absolute-duration family, i.e., variability, dynamics and asymmetry only. This set contains no information about how fast or how slowly the subject walked, only about the temporal structure of the walking.

### 2.5. Classification and Validation

Four-class classifiers were multinomial logistic regression (LR) with L2 and with elastic-net penalties, random forest (500 trees), and gradient-boosted trees (XGBoost, softprob objective). Every model was embedded in a pipeline comprising median imputation, standardisation and the classifier, so that all preprocessing was fitted on training data only. Class imbalance was addressed with balanced class weights (per-sample weights computed within training folds for XGBoost); no resampling was applied outside training folds, since resampling fitted outside the training partition is a known source of optimistic bias [36,37].

Performance was estimated by nested leave-one-subject-out (LOSO) cross-validation: an outer loop of 63 folds each holding out one subject, and an inner stratified 5-fold grid search on the remaining 62 subjects to select hyperparameters (random forest grid: maximum depth ∈ {None, 3, 5} × maximum features ∈ {√*p*, 0.5} × minimum samples per leaf ∈ {1, 2}, scored by negative log-loss). The same scoring rule (negative log-loss on the inner 5-fold split) selected hyperparameters for the other three families, over grids sized for 63 (gaitndd) or 110 (gaitpdb) subjects: L2-penalised logistic regression (lbfgs solver, balanced class weights, max_iter = 5000) searched C ∈ {0.01, 0.1, 1.0, 10.0}; elastic-net logistic regression (saga solver, balanced class weights, max_iter = 8000) searched C ∈ {0.1, 1.0, 10.0} × l1_ratio ∈ {0.15, 0.5, 0.85} (9 combinations); and XGBoost (300 trees, subsample 0.8, column-subsample 0.8, L2 regularisation λ = 1, histogram tree method, softmax/softprob or binary-logistic objective as appropriate) searched max_depth ∈ {2, 3} × min_child_weight ∈ {1, 3} × learning_rate ∈ {0.05, 0.1} (8 combinations), with balanced per-sample weights computed from the training fold only, inside the estimator’s own fit() call, so validation and held-out rows never contributed to the weighting. Across the outer LOSO folds, the modal (most frequently inner-selected) setting was C = 0.1 for L2 logistic regression (matching 98–100% of folds) and max_depth = 3, learning_rate = 0.05 for XGBoost (57–73% of folds on gaitndd); full per-fold selections for every family, feature set and cohort are deposited with the analysis archive (model_hyperparameters.csv/gaitpdb_model_hyperparameters.csv; Data Availability). Out-of-fold predicted probabilities were pooled across all 63 subjects and metrics computed once on the pooled predictions. A repeated stratified 5-fold outer loop over three seeds was run in parallel to characterise fold-to-fold spread, and window-level models were validated with stratified group 5-fold cross-validation grouped by subject, aggregating window probabilities to a subject-level prediction by averaging. Window-level analyses accordingly increase the number of correlated, within-subject time samples available to the classifier, not the number of independent subjects; the unit of statistical inference throughout this study remains the subject (Section 4.3).

Reported metrics are macro-averaged one-vs-rest area under the receiver operating characteristic curve (AUROC), per-class one-vs-rest AUROC, balanced accuracy, macro-F1, per-class precision and recall, and the 4 × 4 confusion matrix. Confidence intervals are subject-level bootstraps (2000 resamples) of the pooled out-of-fold predictions. Contrasts between configurations are paired subject-level bootstraps of the same pooled predictions. All random seeds were fixed and per-fold results retained.

### 2.6. Permutation Test

Because a four-class problem with 63 subjects can produce optimistic cross-validated scores by chance, the best configuration in each feature set was tested against a permutation null. Diagnostic labels were permuted at the subject level and the complete LOSO loop was re-run 1000 times, with preprocessing still refitted inside every training fold. To make 1000 re-runs affordable, hyperparameters were fixed at the modal setting selected by the nested search rather than re-tuned; the observed value used for the *p*-value is the matching fixed-hyperparameter LOSO score, and the nested score is also reported. Empirical *p*-values were computed as *p* = (1 + #{null ≥ observed})/(1 + *n*_perm), so 0.001 is the resolution floor of this test.

### 2.7. Sequence Models

One-dimensional convolutional neural networks (CNNs) and recurrent networks were trained on the window tensors as a comparison model family; each window spans 64 consecutive stride cycles with a 32-cycle (50%) hop between consecutive windows (Section 2.3). Both were kept small given the sample size: the CNN used three convolutional blocks (16, 32 and 32 channels; kernel sizes 5, 5 and 3 respectively, each followed by batch normalisation and ReLU, with max-pooling after the first two blocks only) followed by global average pooling, dropout (*p* = 0.3) and a linear four-class head (6324 trainable parameters); the recurrent model used a single-layer LSTM (hidden size 48) with mean pooling over time, dropout (*p* = 0.3) and a linear head (10,564 trainable parameters). Both architectures were trained with the Adam optimiser (learning rate 1 × 10^−3^, weight decay 1 × 10^−4^, no learning-rate schedule) in mini-batches of 32 windows (batches of size 1 were skipped because of batch normalisation) for a maximum of 150 epochs; layer weights used PyTorch 2.12.1’s built-in default initialisation, since no custom initialisation scheme was set in the training code. Channel normalisation statistics were computed on training windows only. An inner validation split—one of five subject-disjoint folds carved from the training subjects only via StratifiedGroupKFold, i.e., roughly 80/20 within the training partition—was used for early stopping on the validation loss (patience 25 epochs, with the best-validation-loss epoch’s weights restored for evaluation); test windows were never used for early stopping or for any other model-selection decision. Class imbalance was handled with weighted cross-entropy, with class weights computed from training-fold label frequencies only. Each architecture was trained on two input variants: raw z-scored sequences, and detrended sequences in which each window’s per-channel mean was subtracted before z-scoring. This detrending step is a sensitivity analysis conceptually related to, but not mathematically equivalent to, the DECONF feature set: both attempt to remove absolute walking-speed/duration information while retaining within-subject temporal structure, but DECONF drops nine hand-selected absolute-duration summary features from a 37-feature tabular representation, whereas mean-subtraction acts on the raw multivariate time series and additionally removes any other constant offset each channel happens to carry over the 64-cycle window, which is a different and not strictly narrower operation. Results from the detrended sequence models should therefore be read as corroborating evidence that the sequence models’ skill is not solely attributable to absolute-duration information, not as a numerically equivalent counterpart to the DECONF audit. Window predictions were averaged to subject level for reporting. The full cross-validation was repeated over five random seeds (affecting weight initialisation and fold shuffling) to quantify stability, and once under LOSO for the headline figure. Given that this comparison trains multi-layer neural networks on only 63 subjects, with as few as 13 in the smallest class, the CNN and LSTM results reported below (Section 3.2) should be read as an exploratory comparison of model families rather than as evidence of robust generalisation; the fold-to-fold variability documented there reinforces this caveat.

### 2.8. Interpretability

Out-of-fold SHAP values were computed for the best feature model so that no subject’s explanation came from a model that had seen it: for each of the 63 LOSO folds the pipeline was refitted on the other 62 subjects using the hyperparameters the inner search had already selected for that fold, and a tree explainer attributed only the held-out subject’s prediction [38,39]. Refitted fold models reproduced the published out-of-fold probabilities to within 1 × 10^−16^. Global importance was summarised per class as mean absolute SHAP with direction of effect taken from the Spearman correlation between the raw feature value and its signed attribution. Attribution was also aggregated by feature family to quantify how much of each class’s decision rested on the confound-prone family. Top features per class were compared against the gait phenotype expected from the literature for that disease and graded as concordant, neutral or discordant. Local explanations were generated for one typical correctly classified subject per class and for the most confidently misclassified subject.

### 2.9. Confound Audit

Four analyses quantified the demographic contribution.

1. Demographic-only baselines. The identical four-class pipeline and nested LOSO protocol were applied to (i) age alone, (ii) walking speed alone, (iii) age + walking speed, and (iv) age + sex + height + weight + body mass index (BMI). No gait feature entered these models. Missing values were imputed within training folds only (speed for two subjects, weight and BMI for one).
2. Within-fold confound adjustment. Every gait feature was residualised on age and walking speed with the regression fitted on training subjects only and applied to the held-out subject, and the cross-validation re-run.
3. Feature-family ablation. Each family alone, and each leave-one-family-out configuration, was evaluated under the same protocol.
4. Age-matched replication. The four-class analysis was repeated on subjects aged ≥ 40 years (*n* = 47) and on 1:1:1:1 nearest-neighbour age-matched quadruplets (caliper 10 years, *n* = 28, 7 per class).

### 2.10. Model Selection for Demographic Baselines

A demographic baseline is only informative as a comparator if it is given the same opportunity to fit the data as the model it is being compared against. In a first pass we evaluated every demographic block with the random forest used for the gait models, and this produced a systematic bias in our own favour: with one to five continuous predictors and 63 subjects, a depth-limited forest can only make coarse axis-aligned splits, whereas the relationship between age or body size and diagnosis is close to monotone and is captured directly by a regularised linear model. The discrepancy was large enough to change a conclusion—the anthropometric baseline rose from 0.751 (random forest) to 0.812 (elastic-net logistic regression), and the paired-bootstrap contrast against the 37-feature gait model moved from *p* = 0.141 to *p* = 0.701.

We therefore fit every demographic block with the same four model families used for the gait feature sets (L2 logistic regression, elastic-net logistic regression, random forest and gradient boosting) under the identical nested leave-one-subject-out protocol, and report the best-performing family per block, naming it in the results table. Table S1 records all four families for every block in both cohorts, so the selection is auditable rather than a matter of trust. Applying the same rule to the gait feature sets leaves them unchanged, since the random forest was already the best family there.

We note the direction of this correction explicitly: it strengthens the paper’s central claim rather than weakening it, and it was found by us rather than in review. The general point is that an under-powered baseline is not a conservative choice—it is a thumb on the scale, and it points towards whatever the authors set out to show. To make the full selection hierarchy explicit: within each of the four model families, hyperparameters were tuned by an inner grid search nested inside every outer LOSO (or leave-one-site-out) fold, so hyperparameter tuning never saw the held-out subject. The choice among the four families, however, was not wrapped in a further, independent outer layer: for each feature set and cohort we compared the four families’ own pooled nested-LOSO metric—the same metric reported as the headline performance estimate—and named the highest-scoring family as “selected”. This is a coarser, higher-level selection step than hyperparameter tuning, and it is not itself nested; we discuss the resulting risk of optimistic bias in Section 4.4.

### 2.11. External Validation Cohort

To test whether the audit’s conclusion depends on the particular cohort, we repeated it on the PhysioNet Gait in Parkinson’s Disease Database (gaitpdb), an independent public dataset recorded with a different sensor modality. Raw signals are eight vertical ground-reaction-force channels per foot sampled at 100 Hz, rather than the two force-sensitive footswitch channels of gaitndd, so no derived stride-interval series is distributed with the database. We therefore extracted gait events from threshold crossings on the summed force per foot and recomputed the identical 37 features with the same code path, so that the two cohorts differ in data but not in feature definition. The detection threshold was calibrated by matching stance and double-support percentages to the gaitndd control group, a target that is independent of the diagnostic labels; stride interval itself proved insensitive to the threshold across the range tested. The same quality-control rules were applied unchanged, including the transition-step trim and the median-absolute-deviation outlier filter. Of the three recording sites in the database, one (Ju) was excluded in full: its recordings are 40–115 s long against 121 s at the other two sites, which left the nonlinear and spectral features undefined for 25 subjects, 20 of them controls at that single site. Imputing structurally missing values concentrated in one site-by-group cell would inject systematic false values into exactly the kind of cell this paper warns about, so the site was dropped rather than imputed. One further subject with fewer than 64 analysable cycles was excluded, leaving 110 subjects (47 controls, 63 PD) across two sites (Ga 46, Si 64).

Feature comparability across the two modalities was verified before modelling by comparing control groups: 35 of 37 features had a standardised difference below 0.8, including all nine absolute-duration and all ten nonlinear-dynamics features. The two exceptions, stride asymmetry and double-support coefficient of variation, both depend on precise left–right event pairing, which is where the modalities differ most; analyses were run with and without them.

Because gaitpdb poses a binary Control-versus-PD problem, its chance level for macro-averaged one-vs-rest AUROC is 0.500—identical to the four-class internal analysis, since the chance level of a one-vs-rest AUROC is always 0.500 for each class and for their macro average, irrespective of the number of classes (unlike balanced accuracy or overall accuracy, whose chance level is 1/K for K balanced classes; Section 3.2). Raw macro-AUROCs are therefore directly comparable between the two cohorts. We additionally report relative skill, (AUROC − 0.500) divided by (best demographic baseline − 0.500), as a normalised measure of how far a model sits above chance relative to the best demographic-only baseline available in the same cohort, wherever the two are set side by side. The recording site was treated as a candidate confound in its own right and audited both as a predictor of diagnosis and by leave-one-site-out training. Because gaitpdb contains only controls and PD, this cohort can test the PD-versus-control shortcut only: it does not provide external validation of the primary four-class differential-diagnosis task, and the paper’s one positive finding—the HD result—cannot be evaluated in this cohort at all.

### 2.12. Statistical Analysis and Software

Group comparisons used Kruskal–Wallis tests with Holm-adjusted pairwise Mann–Whitney tests, and χ^2^ for sex. Univariate feature screening used Kruskal–Wallis with Benjamini–Hochberg false-discovery-rate control. Analyses were performed in Python 3.11 with NumPy, SciPy, pandas, scikit-learn, XGBoost, SHAP, statsmodels, PyTorch 2.12.1 and the WFDB package. All code, intermediate tables and figure sources are provided (Data Availability).

## 3. Results

### 3.1. Cohort Composition Confirms Substantial Demographic Imbalance

The 63 analysable subjects differed markedly in demographics (Table 1). Controls were much younger than patients (median 35 years, interquartile range (IQR) 22–53, versus PD 68 (58–76), ALS 62 (43–66), HD 47 (38–54); Kruskal–Wallis *p* = 8.4 × 10^−5^; control-versus-PD Holm-adjusted *p* = 0.0013). Walking speed also differed across groups (*p* = 2.9 × 10^−4^), being highest in controls (1.33 m/s) and lowest in PD (0.98 m/s). Sex was imbalanced (*p* = 9.2 × 10^−4^), as was BMI (*p* = 0.020). ALS subjects contributed fewer analysable stride cycles than other groups (median 178 versus 226–232; *p* = 4.4 × 10^−4^), consistent with slower walking within a fixed 300 s recording.

**Figure 1:**
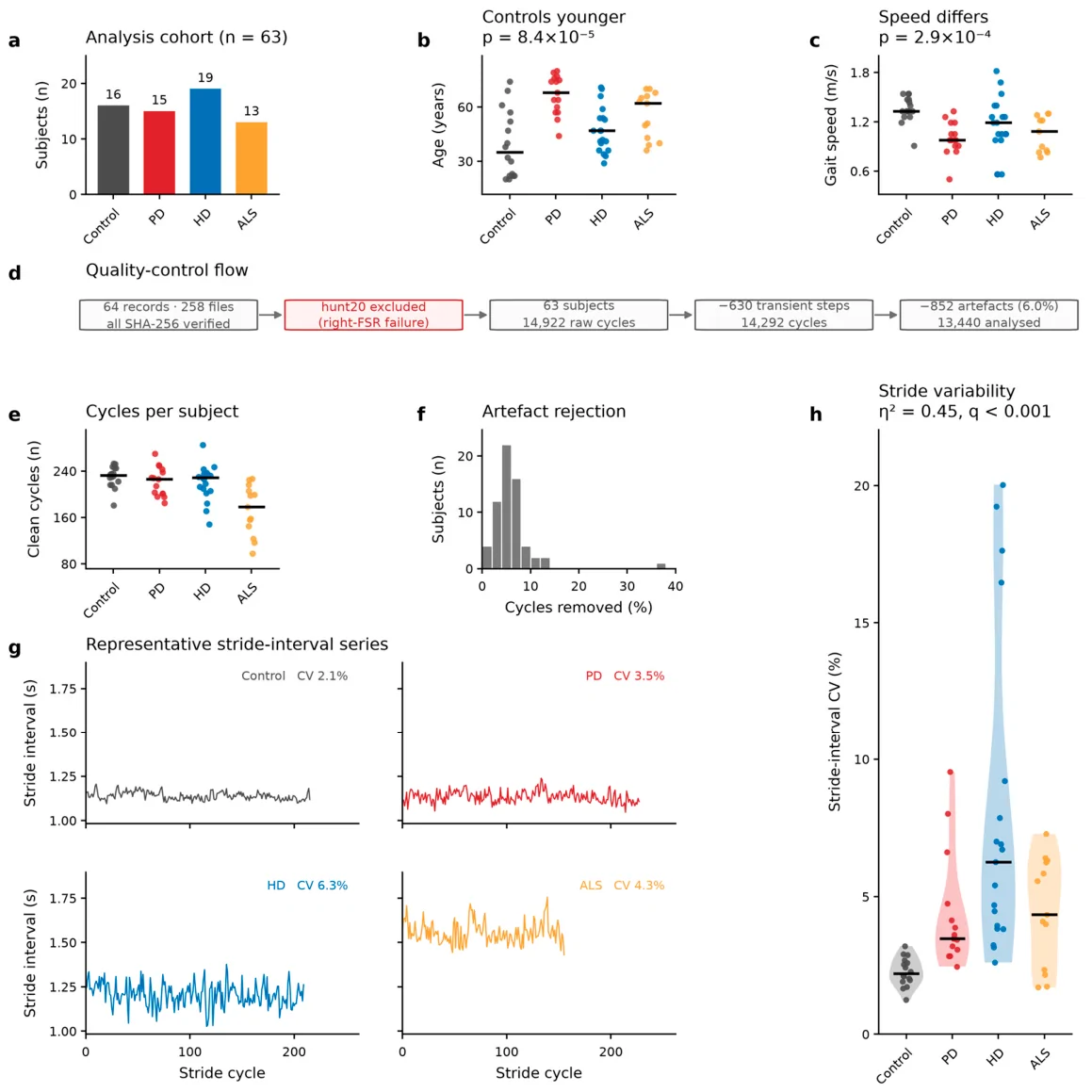
Cohort, demographic confounding and quality control of the gaitndd benchmark. (**a**) Analysis cohort after quality control (*n* = 63: 16 controls, 15 PD, 19 HD, 13 ALS). (**b**) Age by group; horizontal bars are medians. Controls are substantially younger than patients (Kruskal–Wallis *p* = 6.4 × 10^−5^). (**c**) Walking speed by group. (**d**) Quality-control flow from the 64 downloaded records to the 13,440 analysed stride cycles, showing both rejection stages separately: transition-step trimming and artefact rejection. One record (hunt20) was excluded in full because its right-foot force-sensitive resistor failed at source. (**e**) Analysable stride cycles per subject. (**f**) Distribution of the proportion of cycles rejected per subject. (**g**) Representative stride-interval series for one subject per group, each the subject nearest that group’s median stride-interval coefficient of variation. (**h**) Stride-interval coefficient of variation by group, the strongest single univariate marker (η^2^ = 0.45, *q* < 0.001).

Univariate screening reflected the same entanglement: 28 of 37 features discriminated the four groups at a false-discovery rate below 0.05, but 21 of those 28 also correlated with walking speed and 17 with age. The strongest single marker was the stride-interval coefficient of variation (η^2^ = 0.45), which was lowest in controls and highest in HD (Figure 1h).

### 3.2. A Conventional Four-Class Model Performs Well and Is Not a Chance Result

**Figure 2:**
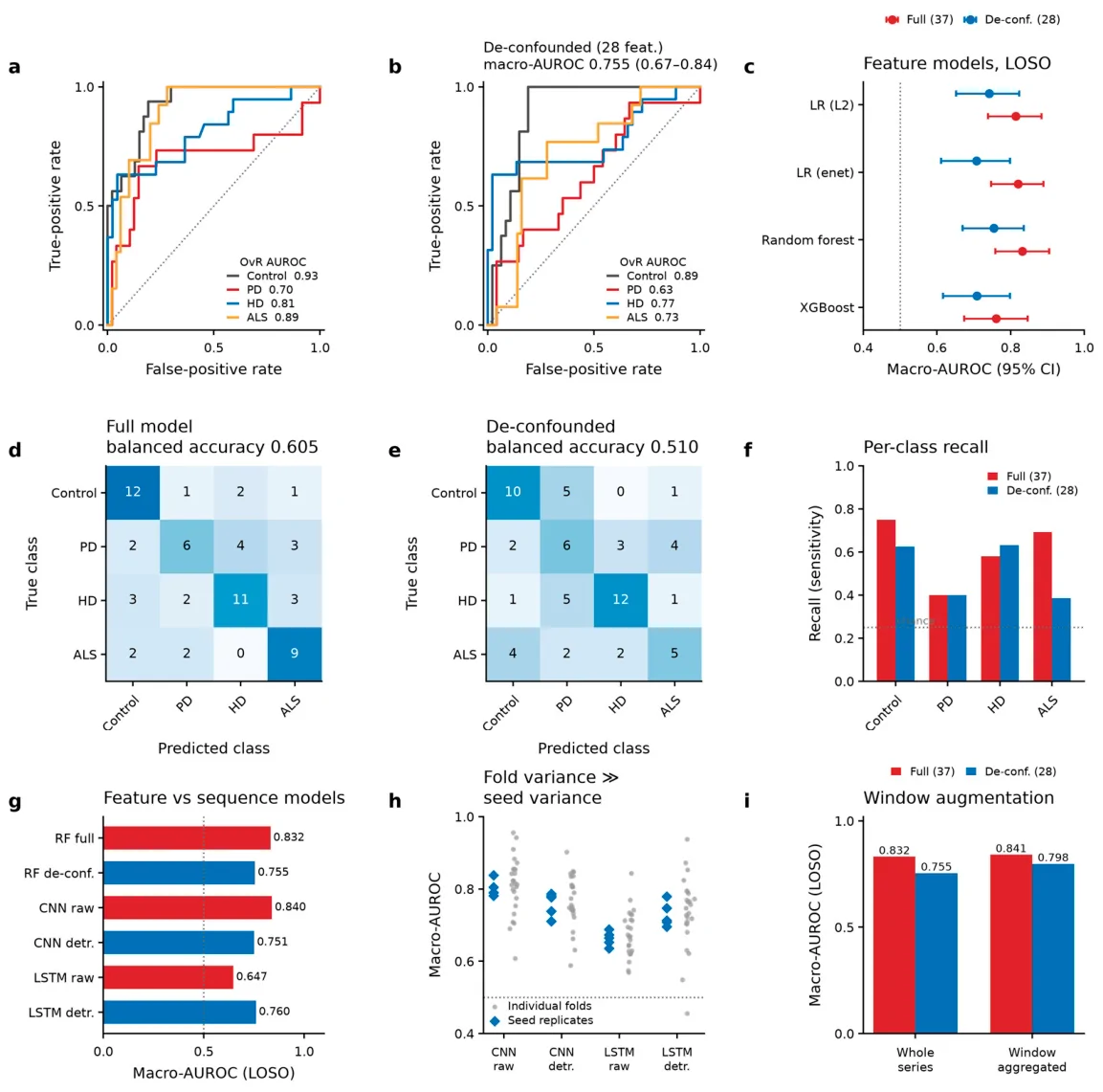
Four-class classification performance on gaitndd. (**a**,**b**) One-vs-rest ROC curves for the best classifier (random forest) on the full 37-feature set and on the de-confounded 28-feature set, computed from pooled out-of-fold predictions. (**c**) Macro-AUROC with bootstrap 95% confidence intervals for all four classifiers and both feature sets. (**d**,**e**) Confusion matrices (counts; shading is row-normalised). (**f**) Per-class recall. (**g**) Feature-based versus sequence models. (**h**) Dispersion across random seeds versus across individual cross-validation folds for the sequence models; fold-to-fold spread exceeds seed-to-seed spread by a factor of 2.5–3.4, so the instability is with respect to which subjects are tested rather than to initialisation. (**i**) Effect of window-level augmentation.

Under nested LOSO cross-validation on all 37 features, the random forest was the best-performing of the four candidate model families within this evaluation procedure (see Section 4.4 for the optimism this induces), reaching macro-AUROC 0.832 (95% CI 0.759–0.904), balanced accuracy 0.605 (0.482–0.725) and macro-F1 0.595 (0.460–0.715). The chance level for a macro-averaged one-vs-rest AUROC is 0.500 regardless of the number of classes, whereas the chance level for balanced accuracy in this four-class problem is 0.25 (Table 2, Figure 2a,c) (the model family itself was selected by comparing this same pooled nested-LOSO metric across four candidates—Methods 2.10; see Limitations 4.4 for the resulting optimism risk). The permutation test placed this far outside the null: the observed macro-AUROC exceeded all 1000 subject-level label permutations (null 0.452 ± 0.072), as did balanced accuracy and macro-F1, so *p* = 0.001 is the resolution floor of the test; the de-confounded model was likewise significant (0.765 against a null of 0.451 ± 0.072). Per-class one-vs-rest AUROC was 0.927 for controls, 0.891 for ALS, 0.812 for HD and 0.699 for PD. PD was the hardest class throughout, with recall 0.40; of 15 PD subjects, 6 were correctly identified, 4 were assigned to HD, 3 to ALS and 2 to control (Figure 2d). PD-versus-HD was the dominant error axis.

**Figure 3:**
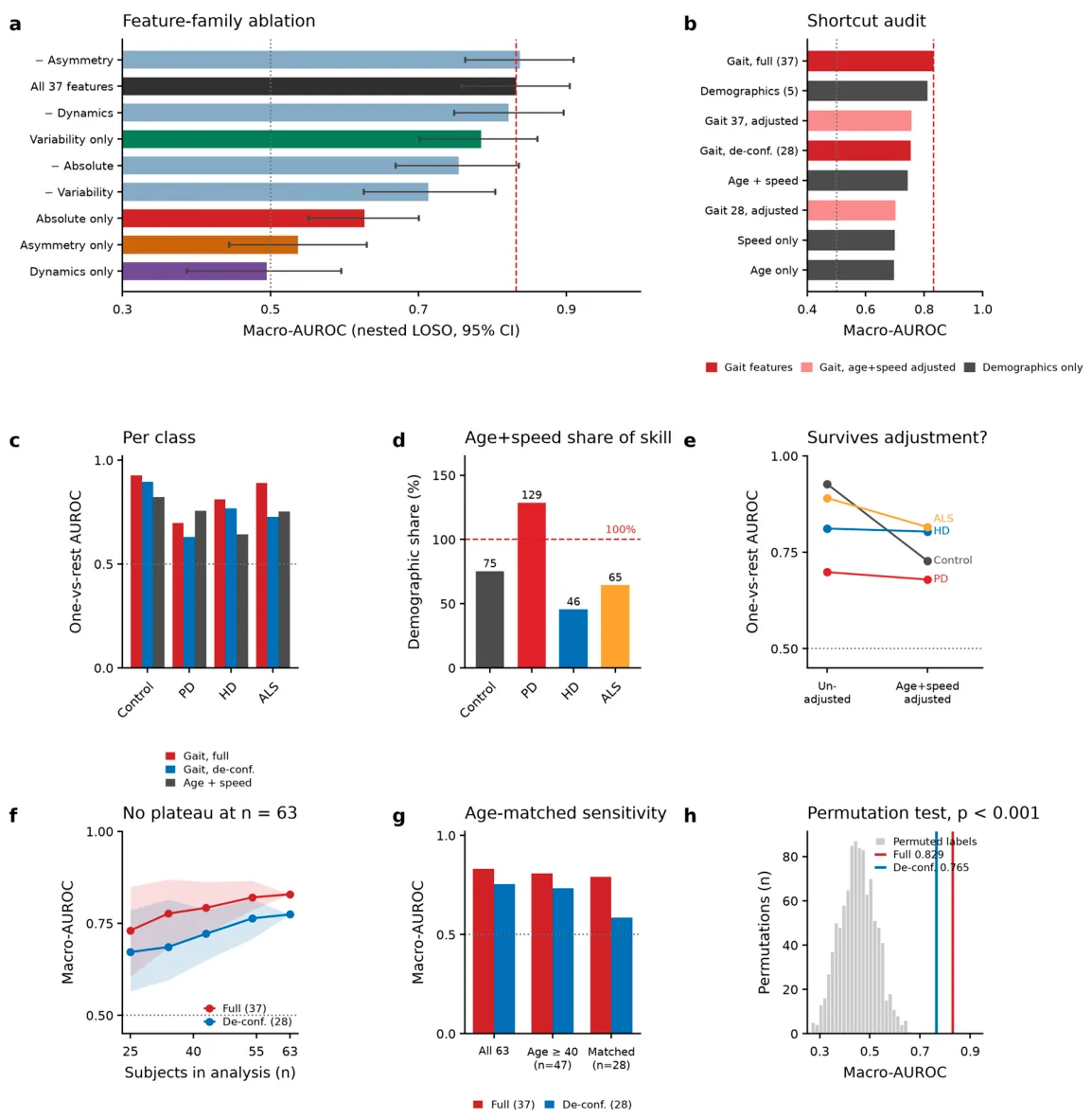
Feature-family ablation, shortcut audit and sensitivity analyses on gaitndd. Every demographic baseline is fitted with the same four model families available to the gait models and the best is reported, so that baselines are not handicapped relative to the models they benchmark (Methods 2.10; the selected family per configuration is listed in Table S1). (**a**) Macro-AUROC for each ablation configuration; the dashed line marks the full 37-feature model. (**b**) Shortcut audit: gait models, confound-adjusted gait models and demographics-only models on one axis. Five anthropometric variables containing no gait information reach 0.812 (95% CI 0.739–0.879), which is not distinguishable from the 37-feature gait model (0.832 (95% CI 0.759–0.904); paired bootstrap Δ = +0.020, *p* = 0.701) and exceeds the de-confounded gait model (0.755 (95% CI 0.669–0.836)). (**c**) Per-class one-vs-rest AUROC for the full gait model, the de-confounded gait model and age plus speed alone. (**d**) Proportion of each class’s above-chance skill attributable to age and walking speed, read off the macro-selected model for each configuration (Table S4); the value exceeds 100% for PD (129%), meaning two numbers a clinician records without instrumentation classify PD better than the 37-feature gait model. HD is the lowest at 46%. (**e**) Per-class AUROC before and after within-fold adjustment for age and speed; HD is nearly unaffected. (**f**) Learning curve by stratified subject subsampling; shading spans the 2.5th–97.5th percentiles across draws. The curve is still rising at *n* = 63. (**g**) Age-matched sensitivity analyses; all bars use a random forest, so the reference is the random-forest full-cohort estimate. (**h**) Permutation null (1000 subject-level label permutations) with the observed values; *p* = 0.001 is the resolution floor of the test.

The observed performance was substantially above the null distribution obtained under this fixed-hyperparameter permutation procedure. Permuting diagnostic labels at the subject level and re-running the entire LOSO loop 1000 times produced a null distribution centred at macro-AUROC 0.452 ± 0.072 with a maximum of 0.666; the observed value exceeded every permutation, giving *p* = 0.001 for macro-AUROC, balanced accuracy and macro-F1 alike, this being the resolution floor at 1000 permutations (Table 3, Figure 3h). The DECONF model was likewise significant (observed 0.765 versus null 0.451 ± 0.073, *p* = 0.001).

Sequence models offered no advantage. A 1D-CNN on raw stride and swing sequences reached subject-level macro-AUROC 0.840 under LOSO, with no statistically significant difference detected from the feature-based random forest at this sample size, while the LSTM on raw sequences performed poorly (0.647) and failed outright on HD (0.379, below chance). Window-level augmentation did not reliably help the best model: aggregating window predictions raised random-forest macro-AUROC from 0.832 to 0.841 for FULL (paired bootstrap Δ = +0.009, 95% CI −0.028 to +0.045, *p* = 0.60) and from 0.755 to 0.798 for DECONF (Δ = +0.044, −0.013 to +0.099, *p* = 0.13); reliable gains occurred only for the weaker learners, indicating variance reduction rather than added information (Table S2, Figure 2i).

A stability analysis of the sequence models exposes a point that applies to the whole study. Seed-to-seed variation was small (macro-AUROC SD 0.019–0.033 across five seeds), but fold-to-fold variation was 2.5–3.4 times larger (SD 0.064–0.100), with individual test folds ranging from 0.456 to 0.956 (Figure 2h). Re-running training reproduces a headline number; changing which subjects are tested does not. The instability of these models is with respect to the sample, not the initialisation.

### 3.3. Demographics Alone Nearly Reproduce the Gait Model

The central result of this study is that models containing no gait data at all perform almost as well as the gait model (Table 3, Figure 3b).

Walking speed alone gave a macro-AUROC of 0.699 (95% CI 0.603–0.786) and age alone 0.697 (0.603–0.784). Age and speed together reached 0.743 (0.651–0.822), and five anthropometric variables—age, sex, height, weight and BMI, with no gait information whatsoever—reached 0.812 (0.739–0.879). Against the 37-feature gait model’s 0.832, neither gap is statistically reliable: every demographic baseline was fitted with the same four model families available to the gait models and the best reported, so the comparison is not handicapped in our favour (Methods 2.10). The paired subject-level bootstrap gives Δ = +0.089 (95% CI −0.017 to +0.190, *p* = 0.097) versus age plus speed, and Δ = +0.020 (−0.086 to +0.120, *p* = 0.701) versus the anthropometric set (Table S3). Five body measurements recover 97% of the gait model’s above-chance skill, and the de-confounded gait model is in fact worse than them (Δ = −0.057, −0.173 to +0.050, *p* = 0.281).

For PD specifically the comparison inverts: age alone achieved a one-vs-rest AUROC of 0.832 and the anthropometric set 0.864, both higher than the 0.699 obtained by the full 37-feature gait model (Table S4). Nothing in the gait signal improved on knowing the subject’s age when the task was to identify PD in this cohort.

### 3.4. Two Independent De-Confounding Strategies Converge on the Same Penalty

Removing the confound costs approximately what the demographic baselines suggest it should, by either route (Figure 3a,b).

Residualising all 37 features on age and walking speed within training folds reduced macro-AUROC from 0.832 to 0.756 (95% CI 0.658–0.848), a loss of 0.076 that the paired bootstrap confirms as reliable (*p* = 0.035). Independently, deleting the nine absolute-duration features—the DECONF set—reduced it to 0.755 (0.669–0.836), a loss of 0.077 (*p* = 0.008); this was the only statistically reliable ablation among all family configurations tested. Two conceptually different de-confounding procedures thus land on essentially the same penalty (0.076 versus 0.077) and the same endpoint (0.756 versus 0.755)—and that endpoint is where the demographics-only models already sit. Consistently, the de-confounded gait model was not better than age plus speed alone (Δ = 0.015, 95% CI −0.092 to +0.123, *p* = 0.86).

The family ablation adds further caution about which features carry information. The variability family alone (12 features) reached 0.785, recovering 94% of the full model’s macro-AUROC, and dropping it cost more (0.713, −0.119) than dropping the absolute family (0.755, −0.077; the only statistically reliable ablation loss, paired bootstrap *p* = 0.008). By contrast the nonlinear-dynamics family alone reached 0.495 and the asymmetry family alone 0.537, neither distinguishable from chance, although both were harmless to retain (dropping asymmetry gave 0.837, nominally the best configuration observed, and dropping dynamics gave 0.822). The absolute family alone reached 0.627, but with HD at 0.298—below chance—showing that absolute duration actively misinforms the HD decision.

### 3.5. SHAP Attribution Localises the Shortcut, and One Misclassification Exemplifies It

**Figure 4:**
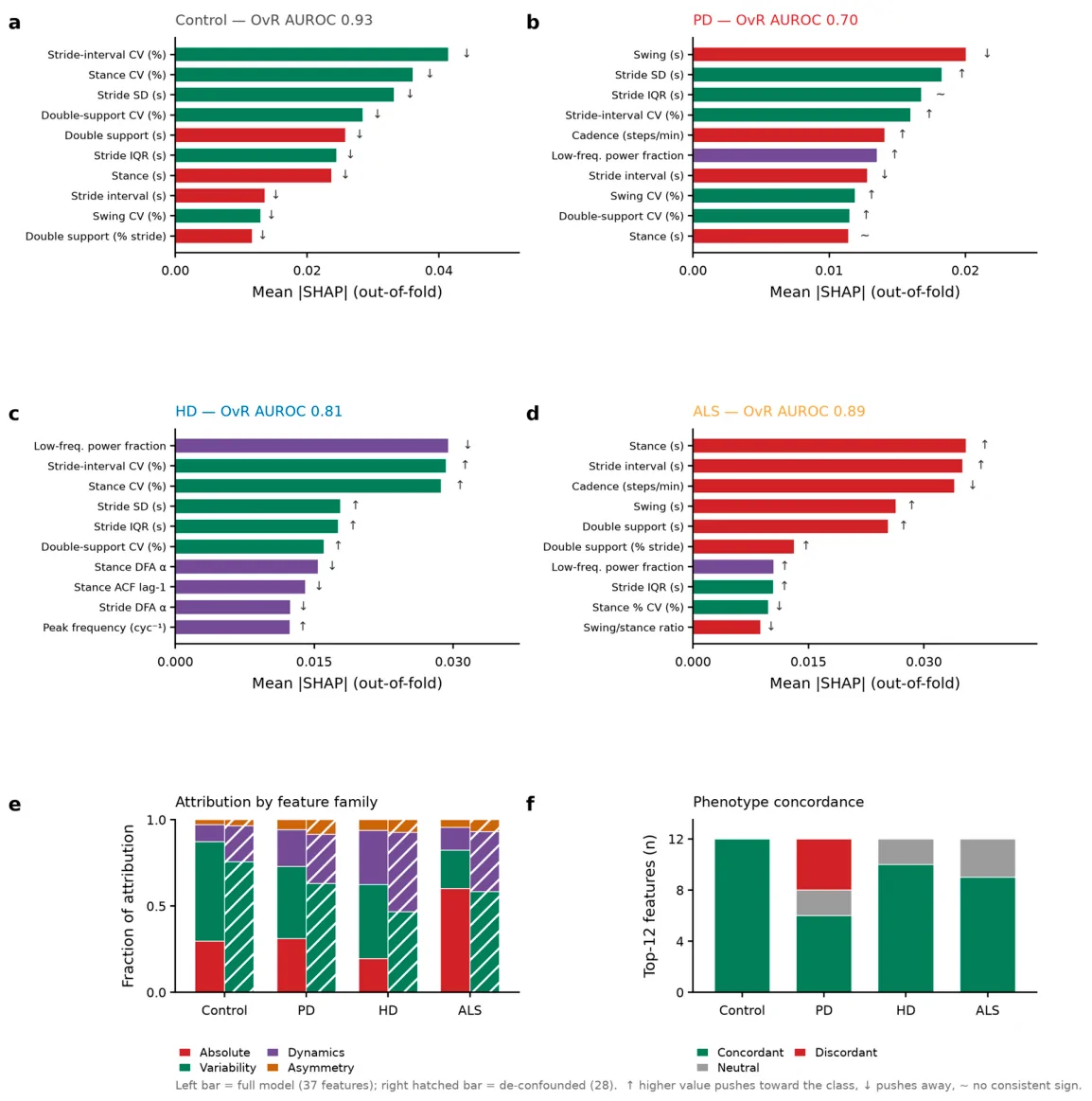
Out-of-fold SHAP interpretation of the full-feature random forest on gaitndd. Explanations are computed out-of-fold, so no subject’s explanation comes from a model that had seen it. (**a**–**d**) Ten highest-attribution features per class, coloured by family; the arrow beside each bar indicates whether a higher feature value pushes toward (↑) or away from (↓) that class, with ~ marking features that carry no consistent sign across subjects. (**e**) Fraction of total attribution carried by each family, per class, for the full model (left bar) and the de-confounded model (right hatched bar). ALS draws 60.2% of its attribution from absolute-duration features. (**f**) Concordance of each class’s top-12 features with the gait phenotype expected from the literature.

Out-of-fold SHAP attribution shows that the classes differ in how much they rely on the confound-prone family (Figure 4e). For ALS, the absolute-duration family carried 60.2% of the attribution; the top five ALS features were mean stance, mean stride interval, cadence, mean swing and mean double support, all duration terms (Figure 4d). For controls the dominant family was variability (57.7%), for PD variability (41.9%) and for HD variability (42.7%) with an unusually high dynamics share (31.4%) and the lowest absolute share of any class (19.6%).

Judged against the gait phenotype expected from the literature, 37 of 41 directionally testable top-feature rows were concordant (90%), and concordant features carried 84.0% of the top-12 attribution mass. Concordance was complete for controls (12/12) and high for HD (10/12) and ALS (9/12), but poor for PD (6/12 concordant, 4 discordant); all four discordances were duration or cadence terms whose sign is set by the four-class contrast against ALS rather than against controls, since ALS subjects in this cohort have the longest intervals, so “shorter stride, faster cadence” reads as PD-like even though PD gait is slower than the control gait (Figure 4f).

**Figure 5:**
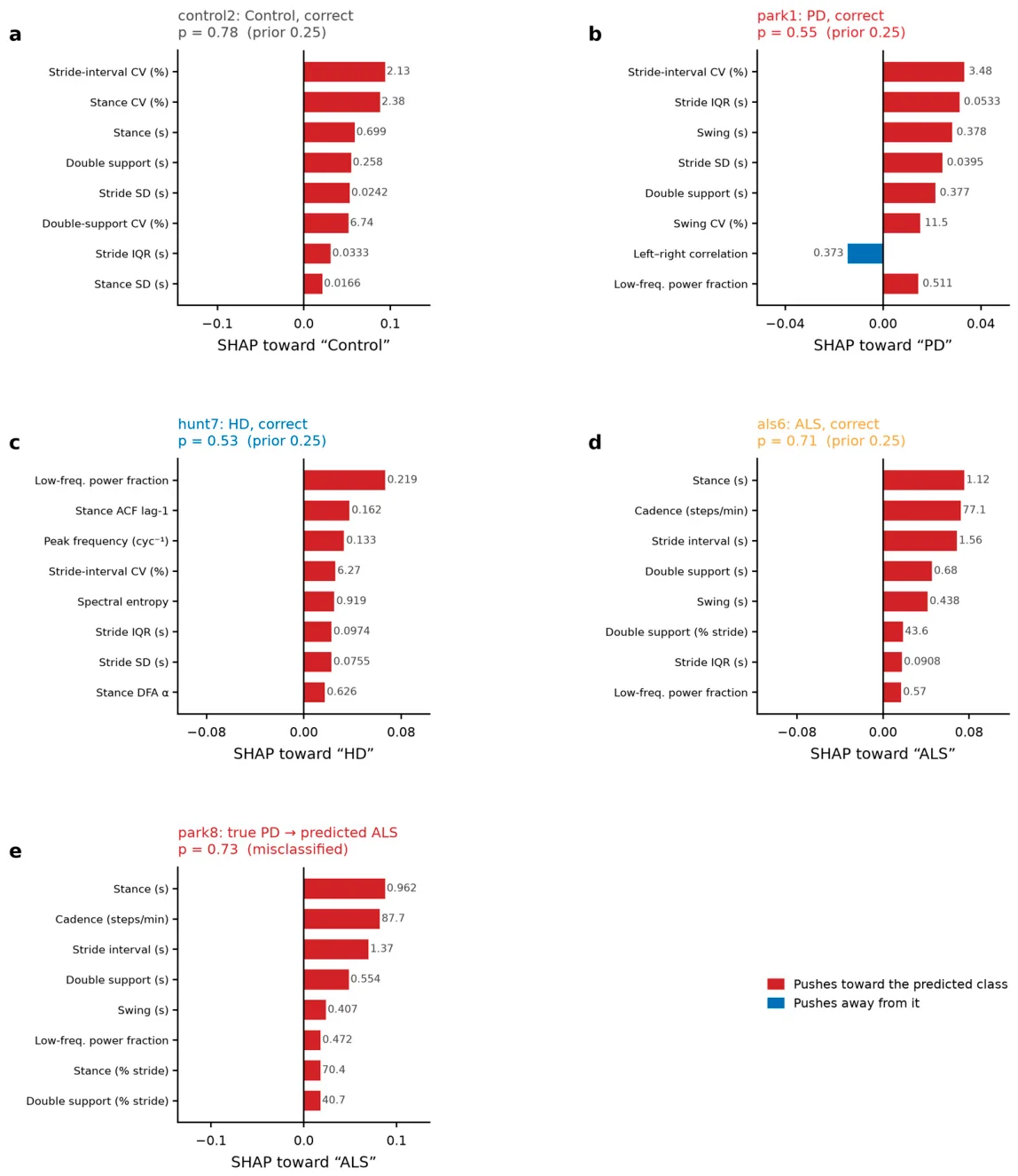
Individual-level SHAP explanations on gaitndd. (**a**–**d**) One typical correctly classified subject per class, each the subject nearest its group’s median stride-interval coefficient of variation. (**e**) The most confidently misclassified subject: park8, true Parkinson’s disease predicted as amyotrophic lateral sclerosis at probability 0.73, driven entirely by absolute-duration features. In all panels, red bars push the prediction toward the predicted class and blue bars push it away (colour key, lower right). Numbers beside each bar are the subject’s raw feature values. Panels (**a**–**d**) are typical correctly classified subjects; panel (**e**) shows that park8’s five strongest drivers are all absolute-duration terms, so the model reads slow walking as amyotrophic lateral sclerosis.

The mechanism is clearest in a single subject. Record park8 (true PD) was predicted ALS with probability 0.734—the most confident error among the 25 misclassifications—and remained ALS in the de-confounded model. Its five strongest attributions toward ALS were all absolute-duration terms: mean stance 0.962 s, cadence 87.7 steps/min, mean stride 1.368 s, mean double support 0.554 s and mean swing 0.407 s (Figure 5e). This subject is a slow, long-interval walker, and the model reads slowness as ALS. The same feature family that achieves a +0.077 macro-AUROC is what makes a slow parkinsonian patient look amyotrophic.

Coupling between predictions and confounds was detectable but confined. Within the 16 true controls, the predicted probability of “control” correlated with walking speed at ρ = +0.637 (*p* = 0.0079; Holm-adjusted *p* = 0.063): the model is more confident that a control is a control the faster that control walks. No other primary coupling reached nominal significance, and misclassification was not significantly associated with age or speed overall (*p* = 0.48 and *p* = 0.31); with 11–19 subjects per class these tests have little power, so the absence of significance is weak evidence (Table S5).

### 3.6. Age Matching Removes the De-Confounded Signal; Performance Has Not Converged

Restricting subjects to those aged ≥ 40 years (*n* = 47, residual age difference still present at *p* = 0.017) left performance close to the full-cohort values: FULL 0.809 (95% CI 0.716–0.893), DECONF 0.733 (0.631–0.833). The headline result is therefore not purely an artefact of the young control group.

Strict 1:1:1:1 nearest-neighbour age matching tells a different story. In 28 subjects with age fully balanced (Kruskal–Wallis *p* = 0.989), the full model survived at 0.791 (0.651–0.915), but the de-confounded model collapsed to 0.585 (0.443–0.715), with ALS falling to 0.286 and PD to 0.469 (Figure 3g). Once age is matched away, the feature set that contains no speed information is no longer distinguishable from chance. This subset discards 35 subjects, and its confidence interval spans 0.26 AUROC units, so it should be read as a sensitivity analysis supporting the direction of the de-confounding effect rather than as quantitative evidence of its magnitude—but it is a sensitivity analysis that points the same way as every other analysis reported here.

Finally, performance has not converged at this sample size. Stratified subject subsampling gave mean FULL macro-AUROC rising monotonically from 0.731 at *n* = 25 through 0.777 (*n* = 34), 0.792 (*n* = 43) and 0.821 (*n* = 54) to 0.829 at *n* = 63, with no flattening, at roughly +0.001 to +0.003 per additional subject over the final intervals (Table S2, Figure 3f). Repetition-to-repetition SD fell from 0.066 to 0.023 but remained of the same order as the entire FULL-versus-DECONF gap (0.077). The reported 0.832 is thus simultaneously a lower bound on what this feature set might achieve with more subjects and an estimate whose subject-sampling variability rivals the effect the study is trying to measure.

### 3.7. Huntington’s Disease Is Most Robust Signal Across Internal Sensitivity Analyses

One signal is robust across all four internal sensitivity analyses performed on this cohort (Table 4, Figure 5c–e), though it has not yet been tested in an independent sample. HD discrimination was 0.812 unadjusted and 0.804 after within-fold residualisation on age and walking speed—a loss of only 0.008, compared with 0.200 for controls. Age and speed together achieved only 0.642 for HD, meaning demographics account for 46% of HD’s above-chance skill versus 75% for controls, 65% for ALS and 129% for PD (Table 4). Removing the absolute-duration family cost HD just 0.044 (14% of its above-chance skill, versus 42% for ALS), and the absolute family alone classified HD at 0.298, below chance. Under strict age matching HD was the only class whose de-confounded discrimination did not deteriorate (0.789 versus 0.768 in the full cohort).

The features responsible are consistent with HD pathophysiology rather than with slowness. The top HD attributions were the fraction of spectral power below 0.1 cycles^−1^ (lower in HD), stride-interval and stance CV (higher), stance and stride DFA α (lower), and stance lag-1 autocorrelation (lower)—that is, high-amplitude, temporally disorganised stepping with degraded long-range correlation (Figure 4c), the pattern described for HD gait in the foundational work on fractal gait dynamics [6]. The sequence models independently corroborate this: detrending the input, which deletes absolute duration, left CNN HD discrimination essentially unchanged (0.788 → 0.762) and repaired the LSTM’s HD failure (0.379 → 0.772), consistent with a recurrent model being distracted by a duration offset it cannot normalise away.

### 3.8. External Validation: The Shortcut Reproduces, but Through a Different Variable

Every analysis above is internal to one cohort, so we repeated the audit on an independent public database with a different sensor modality, different countries of origin and a demographic profile chosen to break one of the two confound pathways. Because gaitpdb contains only controls and PD, this external check is necessarily confined to the binary PD-versus-control shortcut: it does not constitute an external test of the four-class differential-diagnosis task, and the HD finding—the study’s principal positive result—has no counterpart in this cohort and remains externally untested. The gaitpdb cohort comprises 110 analysable subjects (47 controls, 63 PD) recorded with eight-sensor vertical ground-reaction-force plates rather than the force-sensitive footswitches used here, from which we derived the identical 37 features (Section 2.11). Crucially, the two groups are close in age (median 67 versus 63 years, *p* = 0.12) while walking speed remains strongly separated (median 1.08 versus 1.25 m/s, *p* = 5.3 × 10^−6^). The age pathway is therefore largely absent by construction, and the speed pathway is not.

**Figure 6:**
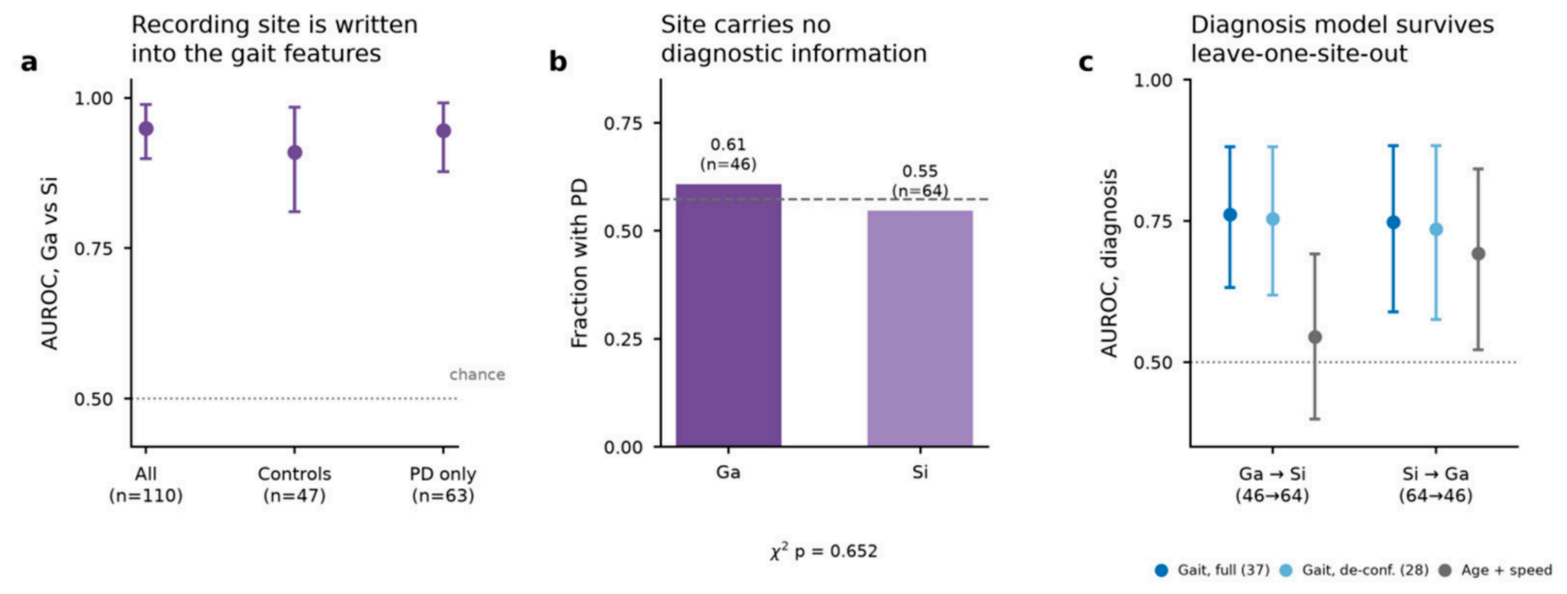
Recording site in the external cohort (gaitpdb; *n* = 110, 47 controls, 63 PD) is a strong feature-level signal but not a diagnostic shortcut. (**a**) Site (Ga versus Si) predicted from the 37 gait features, overall and separately within each diagnostic group; the two recording setups are not exchangeable. (**b**) Proportion with PD by site; the dashed line is the pooled proportion (0.57). Site and diagnosis are independent (χ^2^ *p* = 0.652), and site alone predicts diagnosis at 0.343 (0.241–0.460), below chance. Site could act as a shortcut only because this contingency happens to be balanced; recruitment stratified by site would have manufactured exactly the shortcut this paper warns about. (**c**) Diagnosis model trained on one site and tested on the other, in both directions, with the age-plus-speed baseline shown alongside. In the Ga → Si direction the demographic baseline falls to 0.545 (0.399–0.691), an interval spanning chance, so cross-site transfer of the gait model is not explained by demographics; in the Si → Ga direction the baseline reaches 0.692 (0.522–0.842) and the gap to the gait model is not resolvable at this sample size.

**Figure 7:**
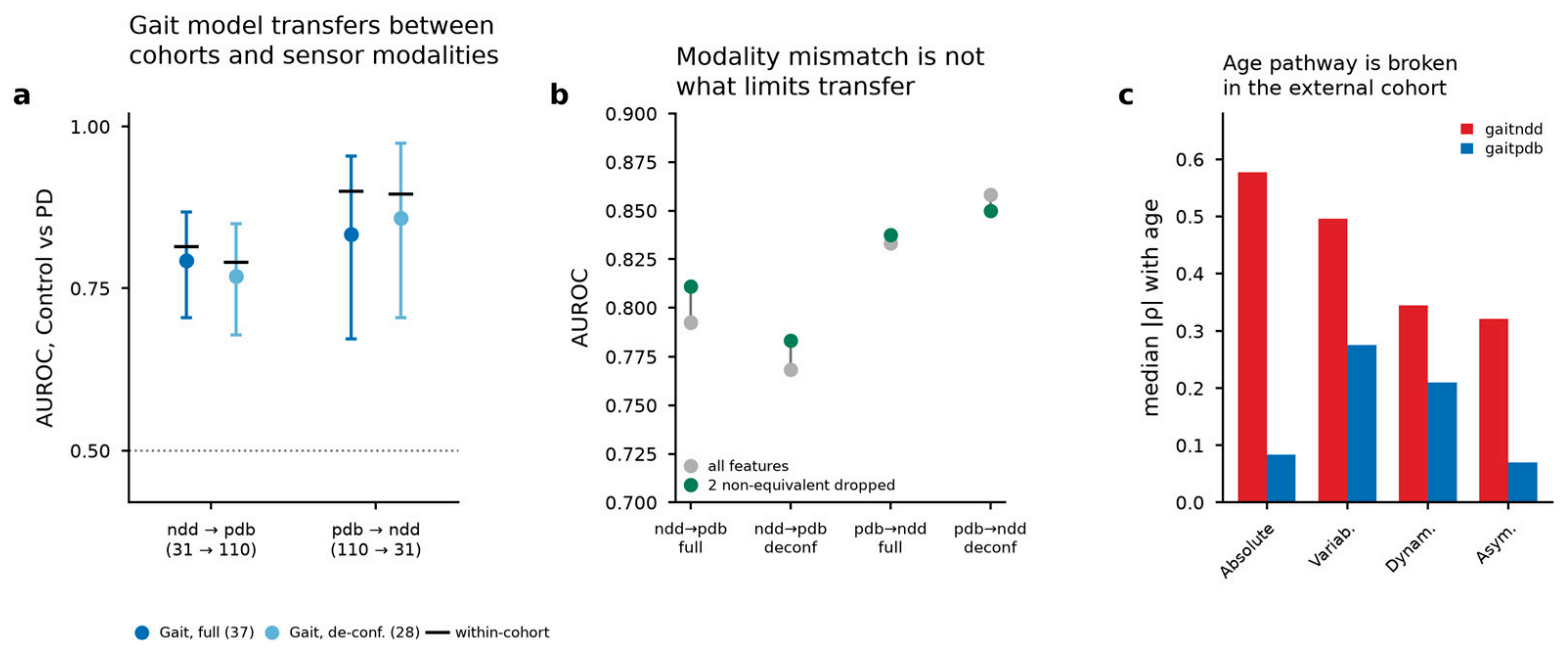
The Control-versus-PD gait representation transports across cohorts, and the demographic pathway carrying the shortcut differs between them. (**a**) Models trained on one cohort and tested on the other, with standardisation and imputation fitted on the training cohort only; black dashes mark the within-test-cohort estimate for reference. (**b**) The same transfers with and without the two features whose distributions are not equivalent across sensor modalities (stride asymmetry, double-support coefficient of variation); dropping them does not hurt transfer, so modality mismatch is not what limits it. (**c**) Median absolute Spearman correlation between each feature family and age, computed with identical code on the Control + PD subset of both cohorts so that the two-class comparison is like-for-like. In gaitndd the absolute-duration family tracks age strongly (median |ρ| = 0.578), whereas in gaitpdb it does not (0.084); the corresponding correlation with walking speed remains high in both (0.717 and 0.606). This dissociation is why the age and speed pathways can be attributed separately across the two cohorts.

Because gaitpdb poses a binary Control-versus-PD problem, its chance-level macro-AUROC is 0.500—the same value as for the four-class internal analysis, since the chance level of a macro-averaged one-vs-rest AUROC is 0.500 regardless of the number of classes. Raw macro-AUROCs are therefore directly comparable between the two cohorts without any renormalisation. We additionally report relative skill, (AUROC − 0.500)/(best demographic baseline − 0.500), as a normalised measure of a model’s skill relative to the best demographic-only baseline available in that cohort. The gait model performed comparably well: an AUROC of 0.828 (95% CI 0.738–0.902), far outside a 1000-permutation null (null 0.495 ± 0.083, *p* = 0.001, again at the resolution floor). The audit then diverged from the internal cohort in exactly the way the cohort’s demographics predict. Age alone reached only 0.545 (0.433–0.663), an interval covering chance, and the five anthropometric variables that were the strongest predictor in gaitndd fell below chance here (0.465). Adding age to speed changed nothing (0.746 → 0.743), and 1:1 age matching within a 5-year caliper (47 pairs, mean absolute age difference 0.94 years) cost only 0.015 AUROC, against the collapse from 0.755 to 0.585 seen internally. Yet the shortcut did not disappear. Walking speed alone reached 0.746 (0.654–0.833), and no statistically significant difference between the 37-feature gait model and walking speed alone was detected at this sample size (Δ = +0.082, 95% CI −0.023 to +0.189, *p* = 0.108), while speed beat age decisively (Δ = +0.201, 95% CI +0.068 to +0.322, *p* = 0.003). Residualising the gait features on age and speed cost a significant 0.087 (*p* = 0.049), confirming genuine dependence on the confound axis. In relative-skill terms the gait model retains 1.33× the skill of a single-variable speed model externally, against 1.06× the skill of the anthropometric baseline internally (recomputed with the corrected AUROC chance level of 0.500; see Methods). Both cohorts therefore land close to their respective demographic baselines, by way of different variables. One internal finding did not replicate, and we flag it as cohort-specific rather than general. The 0.077 penalty for removing the absolute-duration family—our main evidence that this family carries the shortcut in gaitndd—was 0.003 here (0.828 versus 0.825, *p* = 0.892), and out-of-fold SHAP assigned the absolute family only 19.9% of PD attribution against 60.2% for ALS internally. The claim that absolute durations are the carrier is thus specific to gaitndd; what generalises is that a demographic variable substitutes for the gait waveform, not which one. The recording site was audited as a candidate new shortcut and behaved as a domain shift rather than a label leak (Figure 6a–c). Site was highly recoverable from the gait features alone (AUROC 0.949, 0.899–0.988; 0.910 within controls and 0.946 within PD), so the two setups are not exchangeable. But site and diagnosis were independent (χ^2^ *p* = 0.652), and site alone predicted diagnosis at 0.343, below chance, so it cannot act as a shortcut here. That is a property of this cohort’s balance, not a general safeguard: recruitment stratified by site would have manufactured precisely the shortcut this paper warns about. Leave-one-site-out training retained 0.748–0.762 for the gait sets, against 0.545–0.692 for age plus speed. Finally, the representation transferred in both directions across cohorts and sensor modalities: training on the 31 Control + PD subjects of gaitndd and testing on all 110 gaitpdb subjects gave 0.793 (0.705–0.868), and the reverse direction 0.833 (0.672–0.955). Dropping the two features whose distributions are not equivalent across modalities improved the first transfer to 0.811, so modality mismatch is not what limits transfer (Figure 7a,b); the two cohorts also differ in which feature family tracks age, which is where the shortcut enters (Figure 7c). Table 5 lists every external configuration.

## 4. Discussion

### 4.1. Principal Findings

We set out to test, rather than assume, that a gait-based four-class classifier trained on a public neurodegenerative-disease benchmark reads motor pathology. The conventional analysis succeeds on its own terms: a macro-AUROC of 0.832 under strictly subject-disjoint nested cross-validation, with a permutation test that places the result far outside the null. Judged by the standards of much of this literature, that would be the paper.

The audit shows it should not be. Age and walking speed alone reach 0.743; five anthropometric variables reach 0.812. Neither gap from 0.832 is statistically reliable, and the anthropometric model exceeds the de-confounded gait model (0.812 versus 0.755). Removing the confound by within-fold residualisation costs 0.076, and removing the speed-dependent feature family costs 0.077—two independent routes converging on the same endpoint, which is precisely where the demographics-only models already sit, and the de-confounded gait model is statistically indistinguishable from age plus speed. For PD, age alone outperforms all 37 gait features. Under strict age matching the de-confounded model falls to chance-adjacent levels. And the learning curve has not flattened, so none of these estimates has converged.

The interpretability analysis supplies the mechanism rather than merely the magnitude. ALS, the class most damaged by de-confounding, draws 60.2% of its SHAP attribution from absolute-duration features; and the study’s most confident error is a slow parkinsonian walker classified as ALS on stance duration, cadence and stride interval. Interpretability here is not a presentational addition to a performance claim; it is the instrument that identifies which claim is safe to make.

The external cohort settles which part of this generalises. Repeating the whole audit on an independent database with a different sensor modality, in which the two groups are close in age (*p* = 0.12) but still differ in walking speed, reproduces the central result through a different variable: the gait model again fails to separate from a demographic baseline, but that baseline is now walking speed alone (0.746, *p* = 0.108 against the gait model’s 0.828), while age and anthropometry—the operative confounds internally—fall to or below chance. This external test is confined to the binary PD-versus-control comparison, since gaitpdb contains no HD or ALS subjects; it does not validate the four-class task, and it leaves the HD finding—the study’s principal positive result—externally untested. What replicates is that a model using no gait waveform at all comes within 0.02–0.08 AUROC of the full model; what does not replicate is the identity of the substituting variable, or the absolute-duration family’s role as its carrier (0.077 penalty internally versus 0.003 externally, *p* = 0.892). The mechanism is cohort-specific; the vulnerability is not.

Against that, HD was the most robust class across every internal sensitivity analysis performed here, although this robustness has not yet been tested against an independent HD cohort. Its discrimination is essentially unchanged by age and speed adjustment, barely dependent on absolute durations, and actively harmed by them; it is carried by variability and long-range-correlation measures; and it is the one class whose de-confounded performance holds under age matching. This is a positive finding, and it is consistent with the arrhythmic, high-variability stepping with degraded fractal scaling that has been described in HD since the original work on this dataset family [6,7], and with clinical descriptions of HD gait [40,41,42,43].

### 4.2. Relation to Previous Work

Two decades of gait-dynamics research established that stride-interval fluctuations carry disease-relevant structure [5,6,7], in PD [44,45,46,47] as well as in ALS [48,49,50,51,52,53,54]. Our HD result is squarely in that tradition and adds the observation that this particular signal is confound-resistant. Where our findings diverge from much of the recent machine-learning literature on this benchmark [55,56,57,58,59,60,61,62,63,64,65,66] is in the interpretation of multi-class accuracy. High reported accuracies on demographically imbalanced cohorts are compatible with a model that has learned age and speed, and distinguishing the two possibilities requires demographic baselines and de-confounding contrasts that are seldom reported. Our results indicate that on this dataset the distinction matters decisively: the same pipeline that appears to demonstrate four-class differential diagnosis does not outperform two demographic variables once tested against them.

This is an instance of shortcut learning as described in the broader machine-learning literature [28], and it has a specific structural cause in gait research. Because stride, swing, stance and double-support durations are monotone in walking speed, and because both age and disease slow walking, a conventional gait feature set is partly a demographic encoding by construction. The problem is therefore not a defect of a particular dataset or model, and it will not be solved by a larger feature set or a deeper network; it requires either demographically matched cohorts or explicit de-confounding with the resulting performance reported honestly.

### 4.3. Implications

For gait-based classification studies on small cohorts, we suggest that an accuracy claim should be accompanied by four analyses, all of which are inexpensive relative to the modelling itself: a demographic-only baseline using the variables that co-vary with diagnosis; within-fold adjustment for those variables; ablation of the feature family through which they act; and replication in a matched subset. Where a study cannot separate pathology from demographics, that is itself a reportable result—as is the identification of the specific classes and features that do survive.

Two secondary observations may be useful. First, seed-to-seed stability is a misleading reassurance at this sample size, consistent with reports that small-sample validation estimates are dominated by sample composition [29,30,31]: fold-to-fold variation was 2.5–3.4 times larger, so reporting variation across initialisations while cross-validating the same subjects understates uncertainty considerably. Second, window-level augmentation increases the number of correlated, within-subject observations, not the number of independent subjects: the windows are 64-cycle excerpts of each subject’s single walk taken at a 32-cycle (50%) hop (Section 2.3), so consecutive windows from the same subject share most of their underlying strides and are far from statistically independent draws. Consistent with this, aggregating window-level predictions improved only the weaker learners and did not reliably help the best model (Table S2), indicating that the windows mainly reduce prediction variance for a given subject rather than supply additional between-subject information. The effective sample size for every confidence interval, permutation test and power consideration in this study is therefore the number of subjects—63 internally, 110 in gaitpdb—not the number of windows.

For HD specifically, discrimination was essentially unchanged by adjustment for age and speed (0.812 to 0.804) while drawing 42.7% of its attribution from variability and 31.4% from long-range-correlation measures, so these are the more promising candidates for a digital gait marker [13,14], and absolute speed and duration should be reported alongside but not relied upon. Note that this is a class-level result: the HD signal survives adjustment even though individual variability features co-vary with speed, which is why the audit has to be run per class rather than only on the pooled macro-average.

The same distinction matters for longitudinal and rehabilitation monitoring, where a model is applied repeatedly to the same patient rather than once for diagnosis. Because absolute stance, stride and cadence terms carried 60.2% of the attribution for ALS and 31.1% for PD, a classifier weighted towards them tracks how fast the patient is currently walking. An intervention that increases walking speed therefore shifts exactly the inputs these models rely on, and the resulting change in predicted class or class probability would reflect the speed change rather than any change in the underlying disorder. The long-range-correlation and complexity measures are the safer basis for repeated-measures monitoring: none of the ten features in that family correlates with walking speed after correction (median |rho| = 0.08), whereas all twelve variability features do (median |rho| = 0.57), so stride-to-stride variability is itself partly speed-driven and is not confound-free simply because it is not an absolute duration. Any monitoring model built on absolute durations or on variability should be validated against a speed-matched comparison before a change in its output is interpreted as clinical change.

### 4.4. Limitations

The cohort is small (63 subjects; 13 in the smallest class), so every confidence interval is wide, typically 0.15–0.20 macro-AUROC units, and many contrasts are underpowered—”not statistically significant” in this study frequently means “not resolvable at *n* = 63” rather than “no effect”. This cuts both ways: our central negative claim rests on gaps (*p* = 0.097, *p* = 0.701) that additional subjects might resolve in either direction, and our HD claim rests on 19 subjects. A further caveat concerns what the reported confidence intervals do and do not capture. Every interval in this paper is a subject-level bootstrap of the pooled out-of-fold predictions from a single nested-LOSO run (Section 2.5): it propagates sampling variability associated with which subjects happen to appear in a given bootstrap resample, but it does not re-run the inner hyperparameter search, refit the model on the resampled training set, or otherwise change the training-set composition within each bootstrap draw, and so does not propagate the uncertainty introduced by model refitting, by hyperparameter selection, or by model-family selection (see the model-family-selection caveat above) into the width of the interval. This omission plausibly matters here: for the sequence models, where we could compare the two sources directly, re-initialising the network moved the pooled estimate by an SD of only 0.019–0.033 macro-AUROC across five seeds, while evaluating on a different left-out subject moved it by an SD of 0.064–0.100, 2.5–3.4 times larger (Figure 2h). If model-fitting, hyperparameter-selection and training-composition variability behave more like fold-to-fold variability than like seed-to-seed variability—which this fold/seed contrast makes plausible rather than merely a formal possibility—the true sampling uncertainty around every point estimate in this paper is understated by the pooled out-of-fold bootstrap. A fully propagated interval would require re-running the entire nested selection and fitting procedure inside each bootstrap draw, which is outside the scope of this study; we report this as an explicit limitation rather than implying our intervals capture the full uncertainty budget. This constraint applies with particular force to the sequence-model comparison (Section 2.7 and Section 3.2): training a multi-layer CNN or LSTM on 63 subjects, with as few as 13 in the smallest class, is inherently exploratory, and the fold-to-fold AUROC spread of 0.456–0.956 documented for these models should be read as a caution against treating any single deep-learning estimate—including the headline CNN figure—as evidence of robust generalisation.

A related, higher-level source of optimism concerns model-family selection itself. For every feature set and cohort we fitted all four candidate families (L2-penalised and elastic-net logistic regression, random forest, XGBoost) under the identical nested-LOSO protocol and reported the family with the highest pooled out-of-fold metric—the same metric used as the headline performance estimate (Section 2.10). Hyperparameters within each family were properly nested inside every outer fold, but the choice among the four families was not: it compared four already-optimised, non-independent estimates on the same held-out predictions being reported, which is a textbook setting for winner’s-curse-style optimism when the compared candidates are close. In practice the margins between the best and second-best family were modest relative to the bootstrap confidence intervals: 0.011 macro-AUROC (random forest 0.832 vs. elastic-net logistic regression 0.821) on the full gaitndd feature set, 0.013 (0.755 vs. 0.741) on the de-confounded set, 0.001 (XGBoost 0.828 vs. elastic-net 0.827) on the full gaitpdb feature set and 0.024 (L2 logistic regression 0.825 vs. XGBoost 0.801) on the de-confounded gaitpdb set—an order of magnitude smaller than the ~0.15–0.20 AUROC-unit bootstrap interval widths reported throughout this study. Any resulting inflation of the headline estimate is therefore likely small relative to the sampling uncertainty already reported, but it is a real, uncorrected source of optimism: removing it would require an additional outer layer around the family-selection step (e.g., a further nested/double cross-validation), which we did not run because it would add a fourth level of nesting (family × hyperparameter × outer-CV × an additional selection-validation split) that was not computationally tractable within LOSO at *n* = 63/110. Readers should treat the named “best family” and its exact reported score as mildly optimistic rather than as an unbiased estimate of that family’s true out-of-sample performance. The qualitative conclusions of the audit (Section 3.2, Section 3.3, Section 3.4, Section 3.5, Section 3.6, Section 3.7 and Section 3.8) do not depend on which family is named, since every demographic baseline was put through the identical, equally optimistic selection procedure (Section 2.10).

The permutation null and the learning curve used hyperparameters fixed at the modal nested-CV selection rather than full nested re-tuning, which was computationally infeasible at 1000 and 202 re-runs respectively; the offset is small (0.829 versus 0.832 nested) but the values are not exactly comparable to the nested table. The empirical *p*-value floor is 0.001 at 1000 permutations, so *p* = 0.001 should be read as “smaller than this test can resolve”. This fixed-hyperparameter approximation rests on an assumption of hyperparameter stability across outer folds that can be checked directly rather than merely assumed, and the check does not uniformly support it. Across the outer LOSO folds, the modal hyperparameter setting used in the permutation test matched the fold’s own inner-search selection in only 37–38% of folds for the random forest used in the gaitndd permutation test (both feature sets) and 46% for the XGBoost model used in the gaitpdb FULL permutation test; only the L2-logistic-regression model used for gaitpdb DECONF was essentially always at its modal setting (100% of folds), because its 4-point C grid has a single, clearly dominant optimum. Fixing a hyperparameter value that a substantial fraction of folds would not themselves have selected is, in principle, a materially different procedure from the nested search it approximates. In practice, however, the quantity that matters for calibrating the permutation *p*-value—the gap between the fixed-hyperparameter LOSO score and the fully nested LOSO score, on the *true-label* data—was small in every case we checked: about 0.003 (gaitndd random forest, FULL), 0.011 (gaitndd random forest, DECONF), 0.005 (gaitpdb XGBoost, FULL) and exactly 0 (gaitpdb LR-L2, DECONF, by construction). We could not extend this check to the 1000 (gaitndd) or 202 (gaitpdb learning curve) permutation replicates themselves, so we cannot rule out that full re-tuning would shift the *null* distribution by a different amount than it shifts the observed statistic; that would require nested re-tuning inside every replicate, which remained computationally intractable in this study.

The age-matched quadruplet analysis discards 35 of 63 subjects, and with 7 subjects per class a single subject moves per-class AUROC substantially; its confidence interval spans 0.26 units. It should be treated as a sensitivity analysis that supports the direction of the de-confounding effect, not as quantitative evidence of its magnitude.

The external cohort resolves part of this and leaves part open. Because gaitpdb contains only controls and PD, the HD finding—the paper’s one positive result—could not be externally validated; only the PD-versus-control shortcut was tested outside gaitndd, and variability and long-range-correlation measures cannot be proposed as an HD marker until they are replicated in an independent Huntington cohort. The external analysis is also binary, but as established in Section 2.9 and Section 3.2, this does not change its chance level: macro-averaged one-vs-rest AUROC has a chance level of 0.500 regardless of class count, so its estimates are directly comparable to the internal ones on the raw AUROC scale, and relative skill is reported only as an additional, baseline-referenced view.

Within the external cohort itself, three caveats apply. The key contrast—gait model versus walking speed alone—has a 95% confidence interval of −0.023 to +0.189 at *n* = 110, so its non-significance (*p* = 0.108) is an absence of resolution rather than evidence of equivalence. Age matching there used 1:1 greedy nearest-neighbour pairing, and although the mean absolute age difference is 0.94 years the residual is systematically directional, with PD partners older by a mean of 0.60 years (25 pairs PD-older against 4 PD-younger; paired Wilcoxon *p* = 3.5 × 10^−4^); walking speed also remains separated within matched pairs (*p* = 3.3 × 10^−5^), so age matching does not neutralise the speed pathway. And with one of three sites excluded for structurally confounded missingness, leave-one-site-out has only two folds.

Both cohorts share one structural limitation we could not remove: in neither is the demographic confound fully separable from disease. Internally, age and speed differ between groups simultaneously; externally, age is broken but speed is not. A cohort matched on both would be required to attribute the residual gait signal cleanly, and no such public dataset exists for these diseases.

Confound adjustment was linear (residualisation on age and speed). Nonlinear dependence was incompletely removed, so our estimate of the demographic contribution is, if anything, conservative. Walking speed was measured over a separate timed walk rather than derived from the recording, and was missing for three subjects. Disease severity was recorded on group-specific scales that are not comparable across diagnoses, so severity could not be included as a covariate. Finally, the dataset provides a single 300 s walk per subject in one condition, and generalisation to free-living gait, to other sensor modalities, or to earlier disease stages is untested.

### 4.5. Future Work

The four-part audit applied here—a demographic-only baseline, within-fold de-confounding, feature-family ablation, and age-matched or cross-cohort replication—is not specific to this dataset or to gait. It could be applied directly to other publicly available gait and movement-disorder cohorts (e.g., additional parkinsonian, ataxia, or multiple-sclerosis gait databases, and larger multi-site mobility consortia) to test whether the demographic-shortcut pattern identified here is a property of this particular benchmark or a more general feature of small, demographically imbalanced gait studies. Extending the audit to datasets with a wider age range, balanced sex distribution, or matched disease severity would also help separate the confound from genuine biomarker signal in cases where, as here, no single existing cohort permits full separation. The framework generalises beyond gait to any physiological or behavioural signal—voice, keystroke dynamics, wearable-sensor activity, or imaging-derived measures—in which age, sex, or body habitus is known to correlate with both the outcome and the measured signal; in each case, we would expect the same four checks to distinguish a genuine disease signature from a demographic proxy. Finally, because Huntington’s disease could not be tested outside gaitndd, the highest-priority replication is an independent, differently instrumented HD gait cohort large enough to repeat the age-matching and feature-family analyses reported here; absent that, the HD result in this paper should be treated as a single-cohort observation rather than a validated marker.

## 5. Conclusions

A conventional four-class classifier trained on a widely used public gait benchmark reaches a macro-AUROC of 0.832 under strict subject-disjoint validation and is unambiguously better than chance. It is not distinguishable, however, in any statistically reliable sense, from a classifier given only the subject’s age and walking speed, or one given only five body measurements: two independent de-confounding strategies converge on the same performance penalty and the same endpoint, coinciding with the demographics-only baselines. Interpretability analysis localises the mechanism to the absolute-duration feature family, which supplies 60% of the attribution for ALS and drives the study’s most confident misclassification—a slow parkinsonian patient read as amyotrophic. On the evidence assembled here, we therefore do not consider four-class gait-based differential diagnosis to be demonstrated by this dataset.

Huntington’s disease is the exception. Its discrimination is essentially unaffected by adjustment for age and speed (0.812 → 0.804), is carried predominantly by gait variability and long-range-correlation measures rather than absolute timing, and is the only class whose de-confounded discrimination holds under strict age matching—a pattern that held across every internal sensitivity analysis we applied and is consistent with prior descriptions of HD gait arrhythmicity. This robustness is internal to the gaitndd cohort: no external Huntington cohort with a different sensor modality was available to test it, so the finding is best treated as a candidate signal awaiting independent replication rather than an established one.

More broadly, we recommend that accuracy claims from small, demographically imbalanced gait cohorts be reported together with a demographic-only baseline, within-fold confound adjustment, feature-family ablation and age-matched replication. Applied here, that protocol converts an apparently strong multi-class result into a narrower but defensible one. Repeating the demographic-shortcut audit—though not the four-class task itself, which has no counterpart in that cohort—on an independent, binary (PD-versus-control only) cohort recorded with a different sensor modality reproduced the same pattern through a different variable: the gait model was again inseparable from a demographic baseline, but that baseline was walking speed rather than age or body size, and the absolute-duration family carried no penalty there. This is consistent with the underlying vulnerability being general, but two cohorts sharing a data-collection lineage are not sufficient to establish that generality, and the specific mechanism did not transfer. A shortcut audit is therefore a per-cohort requirement rather than a one-time certification that a benchmark passes or fails; Section 4.5 outlines how the same framework could be extended to other gait and movement-disorder datasets to test how general this pattern is. The HD result remains untested outside gaitndd and its independent replication in a dedicated cohort is the necessary next step before it can be considered validated rather than merely internally robust.

## Figures and Tables

**Table 1:** Characteristics of the 63-subject analysis cohort. Continuous variables are median (interquartile range) and were compared with the Kruskal–Wallis test; sex was compared with the χ^2^ test. Controls were significantly younger than patients and walked faster, and ALS subjects contributed fewer analysable stride cycles within the fixed 300 s recording.

Variable	Control (*n* = 16)	PD (*n* = 15)	HD (*n* = 19)	ALS (*n* = 13)	Statistic	*p*
Age (years)	35 (22–53)	68 (58–76)	47 (38–54)	62 (43–66)	*H* = 21.47	8.39 × 10^−5^
Height (m)	1.83 (1.78–1.89)	1.92 (1.72–2.00)	1.83 (1.78–1.90)	1.75 (1.70–1.83)	*H* = 7.52	0.057
Weight (kg)	65.0 (59.0–70.8)	77.0 (62.0–86.0)	75.0 (58.5–86.5)	78.0 (61.2–84.5)	*H* = 3.09	0.377
BMI (kg/m^2^)	20.0 (18.4–21.6)	21.5 (19.5–23.1)	20.6 (18.2–23.7)	24.8 (22.2–26.7)	*H* = 9.83	0.020
Gait speed (m/s)	1.33 (1.31–1.47)	0.98 (0.91–1.12)	1.19 (1.02–1.33)	1.08 (0.84–1.25)	*H* = 18.87	2.91 × 10^−4^
Stride cycles analysed (*n*)	232 (220–245)	226 (201–240)	229 (208–234)	178 (145–206)	*H* = 18.00	4.39 × 10^−4^
Female, *n* (%)	14 (88)	5 (33)	13 (68)	3 (23)	χ^2^ = 16.44	9.21 × 10^−4^

**Table 2:** Four-class classification performance under nested leave-one-subject-out cross-validation, for all four classifiers and both feature sets. Metrics are computed once on out-of-fold predictions pooled across all 63 subjects; confidence intervals are subject-level bootstraps (2000 resamples). The four rightmost columns are one-vs-rest area under the receiver operating characteristic curve (AUROC) per class. The de-confounded set omits the nine absolute-duration features.

Feature Set	Model	Macro-AUROC (95% CI)	Balanced Accuracy	Macro-F1	Control	PD	HD	ALS
Full (37)	Logistic regression (L2)	0.813 (0.738–0.884)	0.586 (0.463–0.708)	0.582 (0.445–0.696)	0.875	0.678	0.819	0.882
Full (37)	Logistic regression (elastic net)	0.821 (0.747–0.889)	0.552 (0.428–0.681)	0.551 (0.416–0.673)	0.870	0.685	0.828	0.900
Full (37)	Random forest	0.832 (0.759–0.904)	0.605 (0.482–0.725)	0.595 (0.460–0.715)	0.927	0.699	0.812	0.891
Full (37)	XGBoost	0.761 (0.674–0.846)	0.554 (0.434–0.676)	0.550 (0.421–0.661)	0.928	0.558	0.762	0.795
De-confounded (28)	Logistic regression (L2)	0.741 (0.652–0.824)	0.479 (0.363–0.599)	0.472 (0.349–0.588)	0.876	0.676	0.774	0.638
De-confounded (28)	Logistic regression (elastic net)	0.707 (0.611–0.798)	0.459 (0.352–0.574)	0.441 (0.321–0.558)	0.892	0.636	0.791	0.508
De-confounded (28)	Random forest	0.755 (0.669–0.836)	0.510 (0.390–0.635)	0.513 (0.386–0.629)	0.895	0.629	0.768	0.726
De-confounded (28)	XGBoost	0.708 (0.616–0.798)	0.473 (0.363–0.592)	0.464 (0.348–0.578)	0.876	0.583	0.727	0.646

**Table 3:** Shortcut audit. All configurations were evaluated with the identical pipeline and nested leave-one-subject-out protocol; demographic-only models contain no gait data, and confound-adjusted models used gait features residualised on age and walking speed with the regression fitted on training subjects only. Every configuration—gait and demographic alike—was fitted with all four model families and the best at the macro level is reported, with that family named (Methods 2.10; the full grid is Table S1). Per-class columns are read off the same macro-selected model, not re-selected per class, which would inflate them by up to 0.083 AUROC (Table S4). Five anthropometric variables reach 0.812 against 0.832 for 37 gait features (paired bootstrap Δ = +0.020, *p* = 0.701) and exceed the de-confounded gait model (0.755; Δ = −0.057, *p* = 0.281).

Configuration	Block	k	Macro-AUROC (95% CI)	Model	Control	PD	HD	ALS
Gait features, all four families (37)	Gait	37	0.832 (0.759–0.904)	RandomForest	0.927	0.699	0.812	0.891
Demographics only: age, sex, height, weight, BMI	Demographics	5	0.812 (0.739–0.879)	LR enet	0.775	0.864	0.672	0.935
Gait 37, residualised on age + speed	Gait, adjusted	37	0.756 (0.658–0.848)	RandomForest	0.727	0.679	0.804	0.815
Gait features, de-confounded (28)	Gait	28	0.755 (0.669–0.836)	RandomForest	0.895	0.629	0.768	0.726
Demographics only: age + gait speed	Demographics	2	0.743 (0.651–0.822)	XGBoost	0.822	0.756	0.642	0.752
Gait 28, residualised on age + speed	Gait, adjusted	28	0.703 (0.602–0.796)	RandomForest	0.770	0.578	0.797	0.666
Demographics only: gait speed	Demographics	1	0.699 (0.603–0.786)	RandomForest	0.789	0.650	0.579	0.778
Demographics only: age	Demographics	1	0.697 (0.603–0.784)	LR l2	0.743	0.832	0.647	0.566

**Table 4:** Per-class decomposition of the shortcut audit. Values are one-vs-rest AUROC read off each configuration’s macro-selected model (Table S4), so a row is internally consistent with Table 3. Huntington’s disease is the only class whose discrimination is essentially unaffected by adjustment for age and walking speed (Δ = +0.008) and whose skill is least attributable to demographics (46%). For PD the demographic share exceeds 100%: age alone reaches 0.832 and five anthropometric variables 0.864, both above the 0.699 of the full 37-feature gait model. ALS is the second most demographically driven class, with the anthropometric set reaching 0.935.

Class	Gait, Full (37)	Gait, De-Conf. (28)	Gait 37, Adjusted	Δ from Adjustment	Age + Speed	Anthropometric	Demographic Share (%)	De-Conf., Age-Matched
Control	0.927	0.895	0.727	+0.199	0.822	0.775	75	0.796
PD	0.699	0.629	0.679	+0.019	0.756	0.864	129	0.469
HD	0.812	0.768	0.804	+0.008	0.642	0.672	46	0.789
ALS	0.891	0.726	0.815	+0.075	0.752	0.935	65	0.286

**Table 5:** External validation on the Gait in Parkinson’s Disease Database (gaitpdb; *n* = 110, 47 controls, 63 PD; two recording sites). This is a binary Control-versus-PD task, its chance-level macro-AUROC (0.500) is the same as for the four-class results in Table 2, Table 3 and Table 4, since one-vs-rest AUROC has a chance level of 0.500 regardless of class count, so raw AUROCs are directly comparable across the two cohorts. Relative skill is (AUROC − 0.500) divided by (0.746 − 0.500), referenced to the strongest demographic baseline (walking speed alone), and is comparable with the corresponding ratio of 1.06 for the internal cohort (anthropometric baseline; Table 3). Every configuration was fitted with all four model families and the best at the macro level is reported (Methods 2.10; the full grid is Table S1). Groups are close in age (*p* = 0.12) but differ in walking speed (*p* = 5.3 × 10^−6^), so the age pathway is largely absent by construction and the speed pathway is not: no statistically significant difference between the gait model and walking speed alone was detected at this sample size (Δ = +0.082, 95% CI −0.023 to +0.189, *p* = 0.108), whereas age and anthropometry fall to or below chance. Removing the absolute-duration family costs 0.003 here (*p* = 0.892) against 0.077 internally, so that family’s role as the shortcut carrier does not replicate.

Configuration	Block	k	AUROC (95% CI)	Model	Relative Skill
Gait features, all four families (37)	Gait	37	0.828 (0.738–0.902)	XGBoost	1.33
Gait features, de-confounded (28)	Gait	28	0.825 (0.737–0.899)	LR l2	1.32
Demographics only: gait speed	Demographics	1	0.746 (0.654–0.833)	LR enet	1.00
Demographics only: age + gait speed	Demographics	2	0.743 (0.651–0.831)	LR enet	0.99
Gait 37, residualised on age + speed	Gait, adjusted	37	0.741 (0.632–0.836)	XGBoost	0.98
Demographics only: age + speed + site	Demographics	3	0.726 (0.633–0.816)	LR enet	0.92
Demographics only: age	Demographics	1	0.545 (0.433–0.663)	LR l2	0.18
Demographics only: age, sex, height, weight, BMI	Demographics	5	0.465 (0.355–0.579)	LR l2	−0.14
Recording site only	Demographics	1	0.343 (0.241–0.460)	LR enet	−0.64

## Data Availability

The two source datasets are publicly available from PhysioNet under the Open Data Commons Attribution License v1.0: the Gait in Neurodegenerative Disease Database (gaitndd, version 1.0.0) at https://physionet.org/content/gaitndd/1.0.0/ (accessed on 5 August 2026) and the Gait in Parkinson’s Disease Database (gaitpdb, version 1.0.0) at https://physionet.org/content/gaitpdb/1.0.0/ (accessed on 5 August 2026). No new human data were generated. All data and code generated by this study are openly available at 10.5281/zenodo.21993343: the analysis code; the derived feature tables; the stride-interval series extracted from the external cohort’s force-plate recordings, the internal cohort’s being recomputed by the deposited quality-control script from the published stride-interval files; out-of-fold predictions and permutation null distributions for every model; the SHAP attributions; and every result table underlying the manuscript and its supplement, including the quality-control logs and the corrected-versus-originally-computed comparisons. Figures are not deposited as images because they appear in the article; the deposited script rebuilds them from those tables. The source recordings are not redistributed, but the download script verifies them against the publisher’s SHA-256 checksums, so every derived file can be regenerated from the public sources.

## Supplementary Materials

The following supporting information can be downloaded at: https://www.mdpi.com/article/10.3390/bioengineering13091092/s1, Table S1: Model-family selection grid for every configuration in both cohorts; Table S2: Subject-subsampling learning curve and the effect of window-level augmentation; Table S3: Subject-level paired bootstrap contrasts between configurations; Table S4: Per-class one-vs-rest AUROC read off the macro-selected model for each configuration; Table S5: Coupling between out-of-fold predictions and the demographic confounds.

## Abbreviations

The following abbreviations are used in this manuscript:

ALS	amyotrophic lateral sclerosis
AUROC	area under the receiver operating characteristic curve
BMI	body mass index
CNN	convolutional neural network
CV	coefficient of variation
DECONF	de-confounded feature set (28 features, absolute-duration family removed)
DFA	detrended fluctuation analysis
FULL	full feature set (37 features)
gaitndd	Gait in Neurodegenerative Disease Database
gaitpdb	Gait in Parkinson’s Disease Database
HD	Huntington’s disease
LOSO	leave-one-subject-out
LR	logistic regression
PD	Parkinson’s disease
RF	random forest
SHAP	Shapley additive explanations
XGBoost	extreme gradient boosting
